# Nocebo-Hypothesis Cognitive Behavioral Therapy (NH-CBT) for Persons With Functional Neurological Symptoms (Motor Type): Design and Implementation of a Randomized Active-Controlled Trial

**DOI:** 10.3389/fneur.2020.586359

**Published:** 2020-12-07

**Authors:** Matt Richardson, Maria Kleinstäuber, Dana Wong

**Affiliations:** ^1^Department of Psychological Medicine, Otago Medical School – Dunedin Campus, University of Otago, Dunedin, New Zealand; ^2^Department of Psychology and Counselling, School of Psychology and Public Health, La Trobe University, Bundoora, VIC, Australia

**Keywords:** functional neurological symptom disorder, psychogenic movement disorder, cognitive behavioral therapy, physical therapy, movement retraining, predictive coding model, randomized controlled trial, nocebo effect

## Abstract

**Introduction:** Functional Neurological Symptom Disorders (FNSD) are associated with high levels of disability and immense direct and indirect health costs. An innovative interdisciplinary rehabilitation approach for individuals with functional neurological symptoms of motor type–Nocebo-Hypothesis Cognitive Behavioral Therapy (NH-CBT)—combines CBT and movement retraining with video feedback embedded in a comprehensive explanatory model of the etiology of FNSD.

**Methods:** This protocol describes the development and implementation of a phase II, parallel group, randomized controlled trial with blinded outcome assessors to compare the efficacy of NH-CBT with an active control condition (supportive counseling and movement retraining). Individuals meeting diagnostic criteria of an FNSD or psychogenic movement disorder will be randomly assigned to one of the 8-week interventions. Self-report scales of motor and other physical symptoms, symptom-related psychological variables, and assessor ratings of participants' mobility will be administered at baseline, and at 8- and 16-week follow-up. Adverse events will be monitored across all sessions and therapeutic alliance will be measured at the end of therapy. The primary statistical analysis will test the hypothesis that NH-CBT is more effective than the control intervention at the 8-week follow-up.

**Discussion:** The therapeutic strategies of NH-CBT are theory-driven by assumptions of the predictive coding model of the etiology of FNSD. Strengths and limitations of this trial will be discussed.

**Trial registration**: Australian New Zealand Clinical Trials Registry (ANZCTR; identifier: ACTRN12620000550909).

## Introduction

Symptoms of altered voluntary motor function that cannot be explained by a neurological disorder are core features of the motor type of Functional Neurological Symptom Disorder (FNSD) (1), or Conversion Disorder as an official term alongside FNSD, and psychogenic movement disorders (PMD) (2). Weakness or paralysis, abnormal movements (e.g., tremor), abnormal gait, and limb posturing are typical motor symptoms. The incidence of persistent functional motor symptoms is estimated to be 4–12/100,000 per year (3). People with functional motor symptoms usually experience severe distress and disability (4), comparable to those with progressive neurodegenerative conditions (5). They are at increased risk of developing comorbid mental health disorders (e.g., major depression, anxiety disorders) (6) and of utilizing health-care resources at high rates (7) resulting in increased direct costs to healthcare systems and indirect costs based on work disability (4).

These epidemiological characteristics highlight the urgent need for effective treatments. FNSD or PMD is one of the most common diagnoses of new referrals to neurological services (8), however over 50% of these individuals do not improve or even feel worse at follow-up (9). There is currently no Class I evidence supporting the efficacy of any treatment for functional motor symptoms (10). The most promising evidence seems to come from cognitive behavioral therapy (CBT) and physiotherapy (11, 12). The central goal of CBT is to identify and modify dysfunctional thinking patterns (e.g. ingrained core thoughts about oneself or one's body, somatic misinterpretations), linked to the motor symptoms, and to promote behavioral changes. For example, typical CBT strategies include psychoeducation (e.g., understanding mechanisms of symptoms), functional behavioral analysis of symptom triggers, taking control of movements, and training of assertive communication (13). In a pilot randomized controlled trial (RCT), participants who underwent CBT tailored for functional motor symptoms significantly improved in daily functioning and secondary outcomes (14). The central goal of physiotherapy is motor reprogramming (15). Usually a systematic strategy is adopted that involves re-establishing elementary movements in affected body regions and reinforcing normal movement, while ignoring abnormal movements. More complex motor tasks are added as soon as individuals are able to perform simple movements appropriately, incrementally approximating normal movement.

Similar to other syndromes of persistent physical symptoms (e.g., chronic pain), mechanisms underlying functional motor symptoms are assumed to be multidimensional and need multidisciplinary interventional approaches. Accordingly, the active ingredients of CBT and physical therapy should be combined into a multidisciplinary approach to optimize outcomes. A consensus recommendation by physiotherapists, neurologists and neuropsychiatrists who are extensively experienced in treating functional motor symptoms, proposed to embed physiotherapy in a biopsychosocial framework (16). This was a first attempt to move toward embedding physiotherapy in psychological (mainly CBT-based), interventions addressing dysfunctional illness beliefs, self-directed attention, psychoeducation, and self-management strategies (16). Non-controlled effectiveness trials of such multidisciplinary treatment programs have demonstrated that combining CBT-based and physiotherapy strategies could be a promising approach for individuals with functional motor symptoms. McCormack et al. (17) and Jacob et al. (18) showed that participants' motor symptoms, mobility, and daily functioning significantly improved. Jordbru et al. (19) combined in a 1-week treatment a physiotherapy intervention following the principles of motor reprogramming (15) and embedded it in psychoeducation and CBT strategies. However, the application of CBT in these studies has generally focused on reducing the stress caused by functional neurological symptoms, rather than altering the underlying beliefs that may be causing the symptoms. Moreover, whilst these previous interventions fit the definition of a multidisciplinary approach (17, 18) or an interdisciplinary approach (19), their strategies do not appear to be integrated around a comprehensive theoretical framework that clearly defines the mechanism behind functional symptoms.

The predictive coding model has become a valuable approach to explain functional motor symptoms (20, 21). This model describes body perceptions as the result of a continuous process of generating, testing, and refining hypotheses of our perceptual system. Learned knowledge about the world is conceptualized as a set of neural representations or “priors” in a hierarchical model. Incoming sensory inputs vary in how well they match with existing priors. The resulting “prediction error” depicts the proportion of input that is not predicted by the prior. Our perceptual system constantly tries to keep this prediction error as low as possible to maintain homeostasis (22). Based on previous experiences a prediction can be more or less “precise” or “strong,” depending on the variance of its distribution. Edwards et al. (20) hypothesizes that functional motor symptoms can emerge based on sensory input (e.g., from a precipitating event or random discharges of neuronal activity) that is afforded excessive precision and strength by attentional processes. The motor symptoms are maintained by the “overweighting of prior beliefs over sensory data.” In accordance with the theory, clinical observations often confirm that distraction can result in a normalization of symptoms. Consequently, the precision of strength of the abnormal prior should be reduced and it should no longer elicit movements when attention is diverted. To our knowledge, there has only one previous treatment been published, a specialist physiotherapy for functional motor symptoms, that is based on the predictive coding model. Nielsen et al. (23) showed moderate effects of this treatment on health-related quality of life in a feasibility RCT.

In contrast to Nielsen et al. (23), we developed an interdisciplinary intervention, Nocebo-Hypothesis Cognitive Behavioral Therapy (NH-CBT), that is unique regarding the way in which elements of psychological and physiotherapy are combined. All disciplines involved in NH-CBT organize their therapeutic input around the direct and transparent addressing of illness beliefs as the primary target for their therapeutic input. The nocebo-hypothesis (NH) part of NH-CBT is proposed to be a crucial concept for the psychoeducation. The concept of a nocebo effect can provide this essential conceptual link between a “prior” and the motor symptom, in a way that most people with FNSD can comprehend. Embedded in the predictive coding model, a nocebo effect is explained as “negative health-related changes in the mind-brain body unit” (24) that are initiated and modulated by expectations (or “priors”). The nocebo effect has originally been studied as a side effect reported in the placebo arm of pharmacotherapy trials (25). Over time the nocebo effect has been extended and has been used to describe adverse effects of stimuli that individuals believe are likely to have negative health effects, e.g., radiation emitted by cell phones (26). Negative expectations can be generated by external sources (e.g., explicit warnings of specific side effects that can occur from a treatment) or just by increased general awareness about the potential of a stimulus to cause health problems (e.g., the belief of an individual that they are especially sensitive to effects of a medication) (26). In people with functional motor symptoms, a subconscious belief that there is neurological damage affecting the function of parts of their body is assumed to be a pathological mechanism. NH-CBT embeds physical interventions in CBT strategies and has the singular aim of disproving the (explicit and/or implicit) belief that the person is neurologically damaged or diseased. In this sense, it follows the traditional CBT's concept of a behavioral experiment, with the “evidence” invariably provided by video feedback in this case.

Compared with previous treatments combining CBT strategies and elements of physiotherapy for functional motor symptoms, NH-CBT has four basic treatment principles that all build on assumptions of the predictive coding model of functional motor symptoms:

- Participants have to understand the role of their “priors” or beliefs as the key mechanism behind their symptoms.
- It is essential to provide the participants with new and precise “motor input” to lower the precision and strength of their abnormal prior beliefs, to develop a new belief, and to help increase the precision and strength of this new belief.
- The precision of the new motor input can be further increased and the abnormal prior belief should be modified by promptly providing participants with visible feedback about their movements.
- Participants' attention has to be diverted to lower its precision-increasing effect on abnormal priors.

Richardson et al. (27) previously examined the effects of NH-CBT in a retrospective case series design without a control group. The authors examined the effect of the treatment protocol in 12 patients with either weakness/reduced mobility, or tremor, or non-epileptic seizures, or mixed symptoms. In seven of the 12 episodes a clinically reliable and significant change of the functional motor symptoms was reached.

The central aim of this RCT is to investigate if NH-CBT for participants with persistent functional motor symptoms is more effective in improving physical functioning (primary outcome) (hypothesis 1) and secondary outcomes (hypothesis 2) in comparison to an active control intervention at the end of therapy. We will also examine if treatment effects can be maintained over 8 weeks post-intervention (hypothesis 3). Participant characteristics at baseline will be investigated as moderator variables of the intervention effect on an exploratory basis.

## Methods and Analysis

### Setting, Participant Recruitment, and Eligibility Criteria

The study will be conducted at the ISIS Centre, a rehabilitation ward of Wakari Hospital (Dunedin, New Zealand), where people with neurological conditions undergo outpatient and inpatient treatment. Participants will be referred by neurologists from the Neurology Department at Dunedin Public Hospital (Southern District Health Board). The ISIS Centre is part of the Southern District Health Board of New Zealand and covers the neurological rehabilitation of 7% of the country's population (~337,000 people).

The inclusion criteria will be:

- Aged 18 or older
- New or returning patients presenting to the participating Neurology Department
- Diagnostic investigations by a neurologist including the appropriate medical examination have been completed.
- Diagnostic investigations resulted in a diagnosis of FNSD with abnormal movement according to diagnostic criteria of DSM-5 (1) or of a PMD according to the diagnostic criteria by Gupta and Lang (2). Acute (<6 months) as well as persistent symptom presentations (≥6 months) will be accepted.
- The symptoms cause clinically significant distress or impairment in social, occupational, or other important areas of daily functioning.

Exclusion criteria will be:

- Diagnosis of a complex regional pain syndrome, Dissociative Identity Disorder, or Posttraumatic Stress Disorder of high severity with significant dissociation
- Severe mental health disorder requiring inpatient mental health treatment or potentially affecting trial participation (e.g., suicidality, acute psychosis, active or extensive self-harm)
- Receiving concomitant psychological intervention that targets motor symptoms
- Initiation or change in regimen of psychopharmacotherapy at any time between 4 weeks before baseline assessment and the final follow-up assessment
- Presence of significant physical trauma/comorbid health conditions (e.g., significant musculoskeletal injuries, severe respiratory illness) precluding participation in our movement retraining (we will include participants who are diagnosed neurological conditions, as long they have individual symptoms that are considered to have a high degree of certainty that they are functional)
- Low English language proficiency
- Inability to provide informed consent to participate in the trial

Functional neurological symptoms are associated with increased rates of comorbid mental health disorders (6). In order to obtain a representative sample, individuals with comorbid mental health conditions (e.g., depression) or other functional (non-motor) symptoms will be included as long as the other condition/symptoms do not require inpatient mental health treatment or potentially affect trial participation (see exclusion criterion b).

### Study Design and Procedure

**Figure 1:**
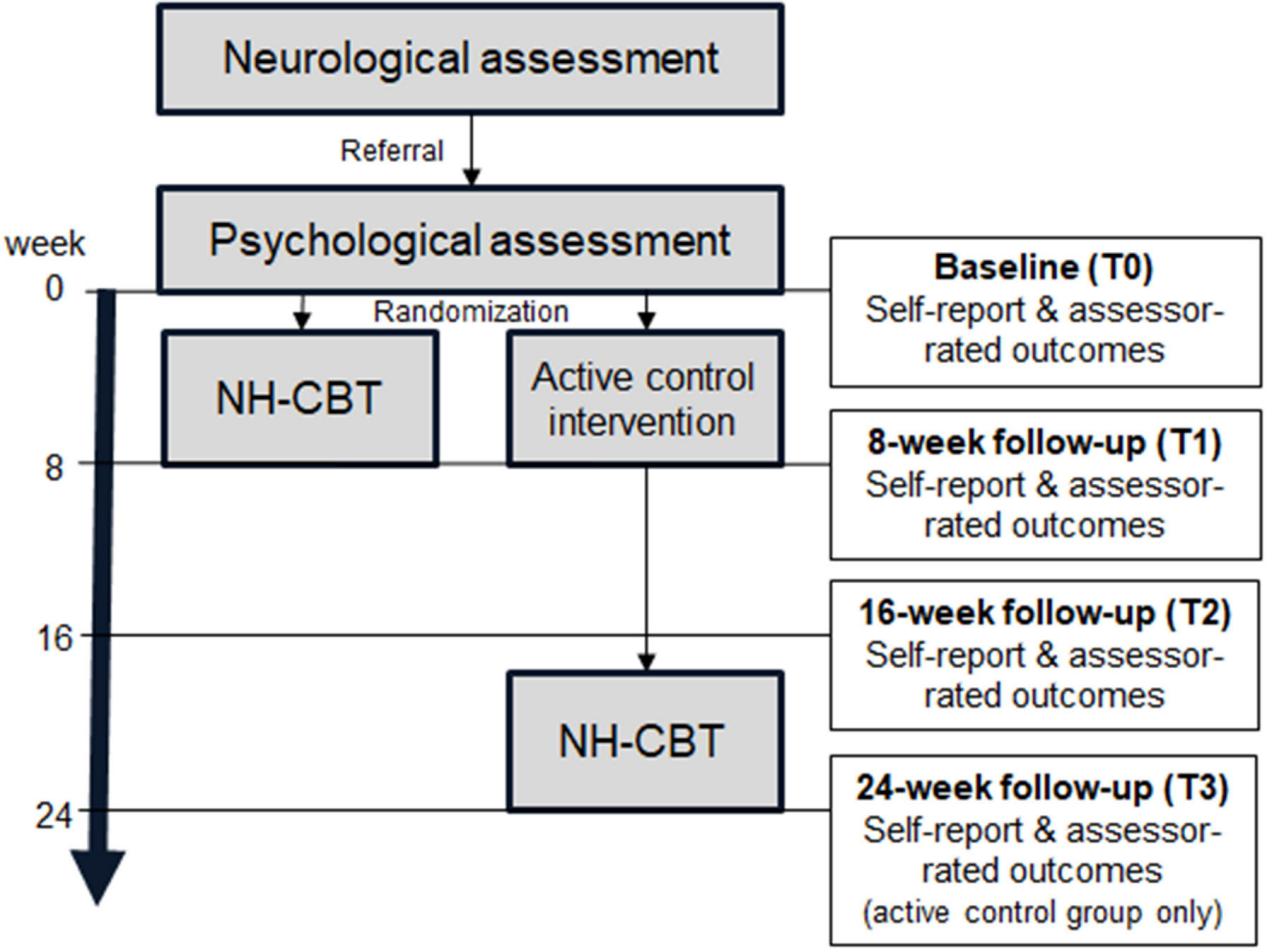
Flow chart of the study procedure and outcome assessments (NH-CBT, Nocebo-Hypothesis Cognitive Behavioral Therapy).

This is a randomized, parallel group, phase II, assessor-blinded, superiority trial comparing the efficacy of a NH-CBT with an active control intervention in individuals with persistent functional neurological symptoms of motor type. Depending on their symptom status at 16-week follow-up, participants in the control group will be offered a delayed NH-CBT treatment. The study procedure is summarized in Figure 1. This protocol has been developed according to the Standard Protocol Items: Recommendations for Interventional Trials (SPIRIT) (see Supplementary Material) (28).

A consultant neurologist will assess participants who are consecutively admitted by the participating Neurology Department using the diagnostic criteria of a FNSD (1) or PMD (2). During the *neurological assessment*, participants will undergo a clinical interview and necessary medical investigations and tests to help establish the diagnosis. The neurological assessment will include an interventional component: the neurologist will give participants a standardized explanation of FNSD/PMD (see *Interventions*). This explanation will follow suggestions by Stone (29). It will include elements such as emphasizing the mechanism of the symptom rather than the cause, explaining how the diagnosis was made, reassuring participants that their symptoms are common, emphasizing the reversibility of their symptoms, and introducing self-help as a key component of a successful treatment. It will be explained how depression and anxiety affect and can perpetuate their motor symptoms. Neurologists will be encouraged to use metaphors as part of their explanations. Individuals who appear to be eligible to participate in the trial will then be informed by the neurologist about the trial. The neurologist will seek participants' permission to be contacted by the principal investigator (MR) to attend the psychological assessment.

The first part of the *psychological assessment* is a clinical interview where the principal investigator (MR) will provide individuals interested in participating with more detailed information about the study. We will explain to potential participants that we will offer them one of two treatments: one is conventional and more commonly used for individuals with FNSD, and has been found to be effective; and the other is new, and we are yet to establish evidence of its efficacy. Participants will be blinded to details of the content and proposed mechanisms of NH-CBT and the control treatment, and to differences between both treatments. They will only be informed that the core component of both treatments will be movement retraining, delivered by a rehabilitation professional. During the first part of the psychological assessment we will assess whether individuals meet all eligibility criteria and will ask them to give written consent to participate. In the second part, an assessor who is independent of this study and trained in applying the assessment tools will do the *baseline assessment*. The research assistant will do the Simplified Functional Movement Disorder Rating Scale (S-FMDRS) (30), the Functional Mobility Scale (FMS) (31) and the 10 m-Walk Test (32) with the participant. A physiotherapist will be present to ensure the safety of the participant. Participants will complete a baseline questionnaire including questions about demographic variables and all other outcome measures (see *Outcomes and Assessments*).

Participants will then be randomly assigned to receive either NH-CBT or the active control treatment over the following 8 weeks. At the end of the intervention period (*8-week follow-up*) participants will attend another assessment with an independent, blinded assessor and will complete all outcome measures. Sixteen weeks after baseline, participants from both groups will complete all outcome measures again (*16-week follow-up*). At that point, participants in the control group who have not achieved symptom remission will be offered NH-CBT and will be asked to complete a final questionnaire after 8 weeks of treatment (*24-week follow-up*).

The authors have planned qualitative interviews at the post-therapy stage with the first 10 patients from either arm of the trial, plus all patients identifying as Māori, to elucidate participants' experiences of their treatment and understanding of their symptoms, and to investigate the acceptability and feasibility of the trial processes and cultural influences on patients perception of treatment.

### Methods for Minimizing Bias

To minimize *selection bias* eligible participants will be randomly assigned to either the NH-CBT or the active control group in a 1:1 ratio using a block randomization strategy (8 blocks with 20 and 1 block with 12 participants). Randomization will be conducted by a research associate not affiliated with the study. The randomization scheme will be generated by using the website randomization.com which applies a pseudorandom number algorithm (http://www.randomization.com). Group allocation will be provided in sealed opaque sequentially numbered envelopes. The principal investigator (MR) and therapists involved in the study will not be masked to the results of the randomization, however the outcome assessors will be blinded to minimize *detection bias*. Before each of the follow-up interviews, participants will be clearly instructed not to discuss treatment content or the identity of their therapists with their assessor. After each follow-up assessment, the blinding of the outcome assessor will be checked with several self-report items (e.g., “Please guess which condition the participant was in”). In the case that the blinding of an outcome assessor cannot be ensured, the three assessor-rated measures will be re-done by a fully blinded assessor. Individual rehabilitation staff will deliver only one of the interventions, either NH-CBT or the control treatment, to reduce the risk of *contamination bias*. Participants from different treatment groups will not be treated in the same room/gym at the same time. Most of our participants will undergo an outpatient treatment and it will be less likely that we have several patients with FNSD receiving inpatient treatment at the same time. It is therefore less likely that participants will communicate with each other about the course of their treatment. Inpatient participants will be instructed not to discuss the details of their treatment with other patients, and inpatient rehabilitation staff will be instructed to remind inpatients of this. To minimize *reporting bias* the study is registered and all pre-specified outcomes are published under the Australian New Zealand Clinical Trials Registry (ANZCTR; identifier: ACTRN12620000550909). To minimize *bias due to loss to follow-up* participants will be sent text reminders on the morning of their appointments. Participants will be considered a dropout when they miss more than two sessions without calling first. Travel costs are not provided directly by the trial but can be reimbursed by other agencies under certain circumstances. Participants will be supported in accessing these reimbursements when eligible.

### Data and Patients' Safety Monitoring

Participants with any type of neurological diagnosis with a range of associated physical disabilities (e.g., weakness, balance difficulties) are at some risk of falling. According to clinical observations, people with FNSD/PMD are at lower risk of injury than people with neurological damage (16). However, there are still some incidents with this group. Treatment necessarily involves taking managed risks whilst ensuring safety, using management strategies and mobility aids. All trial participants will be assessed by a physiotherapist as they start the movement retraining part of the treatment (see *Interventions*) and throughout the treatment if needed, to ensure adequate steps are taken to ensure safety. In the case of serious adverse events (SAE) the Severity Assessment Code (SAC) rating for adverse event reporting (33) will be completed by the therapist. The study therapist has to contact the principal investigator (MR) within 24 h after the event. The PI will not be blinded to the result of the randomization and will therefore be able to decide if the SAE was a serious adverse reaction to the trial treatment.

In order to monitor and supervise the progress of the trial and SAEs associated with the trial treatment, an independent Data Safety and Monitoring Board will be involved, including a psychiatrist, a statistician, a manager of the ISIS Centre and a person who was formerly treated for FNSD at ISIS as a stakeholder of patients' interests. The DSMB will meet on a regular basis and will make final decisions to terminate the trial prematurely for harm, based on their monitoring and evaluation of SAE attributed to our treatment. The SAC rating for adverse event reporting allows for differentiation of four levels of severity of adverse events (34). In the case of severe and major events (SAC 1 and 2), the trial would have to be suspended with immediate effect pending investigation. The DSMB will discuss SAC 3 and 4 events (minor to moderate events), how they relate to our treatment and how they will influence the further course of this trial. The DSMB will also make final decisions to terminate the trial prematurely for benefit, based on stopping rules and interim analyses described below (see *Sample size, power calculations, and interim analyses*).

### Interventions

#### Structure, Providers, and Treatment Fidelity

Participants in both study groups will first receive an explanation of their functional motor symptoms by their neurologist, as part of the *neurological assessment* (described earlier under *Study Design and Procedure*). The main part of the intervention in both groups will start with an initial 60–90 min session that includes education of participants. In both groups, this initial session will be followed by a minimum of 2 up to a maximum of 23 sessions of movement retraining and one final session of relapse prevention. Differences between the groups in the content of the initial session, the movement retraining and the relapse prevention are described below. Sessions will last between 30 and 120 min and will be delivered face to face over 8 weeks. The frequency and scheduling will depend on participants' individual needs (e.g., type and severity of symptoms) and stamina. (see Criteria for finishing the movement retraining before a maximum of 23 sessions can be found in Table 1 under “8. When and how much”. Sessions will follow a treatment manual and will take place either as an inpatient or an outpatient treatment. For both interventions, the initial education session will be conducted by a licensed clinical psychologist, and the movement retraining will be delivered by a licensed rehabilitation professional (physiotherapist, occupational therapist, rehabilitation assistant), but will necessarily involve physiotherapist oversight and involvement at some point. Gender, age, professional backgrounds, and years of experiences of the neurologists, clinical psychologists, and rehabilitation professionals delivering the NH-CBT and the control intervention will be recorded in the main trial paper. Table 1 outlines details of Nocebo-Hypothesis Cognitive Behavioral Therapy (NH-CBT) and the active control treatment (supportive counseling and movement retraining), as well as the means of ensuring treatment fidelity, according to the TIDieR checklist (37).

**Table 1:** Nocebo-Hypothesis Cognitive Behavioral Therapy and supportive counseling and movement retraining (active control intervention) described following the TIDieR checklist.

**Items TIDieR checklist**		
1. Name	Nocebo-Hypothesis Cognitive Behavioral Therapy (NH-CBT)	Supportive counseling and movement retraining
2. Why	The current intervention is based on a predictive coding theory of FNSD/PMD. This theory highlights the following key mechanisms of functional motor symptoms: Pre-disposing factors (e.g., past events experienced or witnessed that lead the person to doubt the integrity of their neurological system) lead to a mental representation of their body/parts of their body to be neurologically damaged or vulnerable to neurological damage, dysfunction or disease. When triggered by a specific situation (e.g., that strongly confirms that belief), neurological functioning starts to change creating physical symptoms.	The control intervention is designed to control for the non-specific effects of (a) education about symptoms, (b) emotional support from a therapist, and (c) movement retraining. The intervention is based on a model of sequential motor learning (16). This model assumes that movement can be retrained or “reprogrammed” by utilizing a sequential approach to re-establish more normal movement patterns.
3. What (materials)	*Information for Neurologists:* Neurologists will receive written information about a standardized explanation of FNSD/PMD, following suggestions by Stone (29) (emphasizing the mechanism of the symptom rather the cause, explaining how the diagnosis was made, emphasizing that it is common, emphasizing reversibility, that self-help is essential for recovery, the possible effect of depression and anxiety on symptoms, and the use of metaphors).	
	*Worksheet*: Written information about the diagnosis and treatment will be provided to participants in the initial educational intervention, to supplement the verbal explanations. This sheet will outline the “nocebo hypothesis” regarding functional symptoms, including information about the concept of subconscious processing and the placebo/nocebo effect, as well as affirming much of the information given by the neurologists. The description of the treatment will deliver more information about the movement retraining, including video feedback.	*Worksheet*: Written information about the diagnosis and treatment will be provided to participants in the initial educational intervention, to supplement the verbal explanations. This sheet will contain information similar to that outlined by the neurologists (see TIDieR Checklist item 3), as well as the basic information about the physical therapy component of the treatment program.
	*Intervention manual*: Each therapist in the experimental condition will receive a NH-CBT manual that complements the NH-CBT training and contains background information about FNSD/PMD, a guide to using the treatment manual, and a detailed description of each of the treatment components with examples of wording.	*Intervention manual*: Each therapist in the control condition will receive a manual describing the supportive counseling and movement retraining, containing a guide to using the treatment manual, a detailed description of each of the treatment components with examples of wording, and information about what content should be avoided (e.g., explaining the nocebo effect and the role of beliefs as mechanisms of the symptoms).
	Portable electromyography biofeedback unit and tablet to provide video feedback	Since the control intervention does not include video feedback or any other form of visual feedback, no tablet or electromyography biofeedback unit is provided.
	Additional materials such as mobility aids (e.g., walking frame), parallel bars, treadmill, etc. may be required depending on the participant's needs	
4. What (procedures)	*Neurological assessment*: Participants in both study groups will be assessed by a neurologist prior to be enrolled in the trial. The neurologists will provide the participant with a standardized explanation of FNSD/PMD (see TIDieR Checklist item 3).	
	*NH-CBT psychoeducation (1 session)*: *Understanding the diagnostic evidence*: Therapists are asked to study participants' health records to gain objective evidence for the FNSD/PMD diagnosis and to identify any historical reasons why someone might believe that they are susceptible to the symptoms they are presenting with (including past medical events, the onset, and course of symptoms). *Assessment:* The main goal of this part of NH-CBT is to explore pre-disposing factors and triggers of functional motor symptoms from participants' subjective point of view. *Transparent sharing of the nocebo hypothesis:* This treatment component addresses participants' personal beliefs about the causes of their symptoms, and their understanding of the terms “subconscious” and “placebo/nocebo effect” (using a graphical aid and metaphors to explain the effect of expectations and belief that a medication works) is explored. A psychological formulation is shared with the participants that incorporates presenting motor symptoms within a hypothesis that symptoms are due to a “nocebo effect.” The aim is to engender in the participant an alternative belief about their symptoms, to challenge the one currently held. The therapist rounds up this component with framing the role and goals of the movement retraining within the nocebo hypothesis.	*Psychological input and supportive counseling (1 session)*: The first session of the active control intervention includes elements of a treatment protocol by Nielsen et al. (30). *Assessment:* The participant's symptom history is assessed regarding current symptoms and health-related problems, symptom onset, predisposing/precipitating/perpetuating factors, symptoms' impact on daily life, previous experiences with treatments and health care professionals, and exploration of participants' understanding of their symptoms and their cause. *Education:* The therapist reiterates the education about FNSD/PMD that was given earlier by the diagnosing neurologist according to the suggestions by Stone (29). The therapist will ensure that the person knows that symptoms are not caused by structural damage or a degenerative process. Then a brief symptom model of FNSD/PMD is provided that conceptualizes functional motor symptoms as a result of a fight or flight (survival) response and secondary behavioral changes (e.g., avoidance behaviors) causing disability. *Supportive counseling*: The therapist uses active listening and supportive counseling skills (e.g., conversation about coping strategies, impact of symptoms on family, and work) but should not adopt a structured therapy approach.
	*NH-CBT movement retraining with video feedback (2–23 sessions)*: The participant is then given opportunities to physically experience themselves as functioning better. The movement retraining is individualized, according to participants' symptoms and needs. The intervention manual proposes exercises for different forms of functional motor symptoms (e.g., functional weakness, balance, tremor, abnormal gait/jerks/myoclonus/involuntary movements, or fixed dystonia). The exercises function as behavioral experiments, to demonstrate to participants that their brains and bodies are capable of working better/normally under certain circumstances. This variability is interpreted to be almost always a sign that there is no damage or disease causing the symptoms. Therapist and participants repeatedly reflect that any improvements can only be due to a change of belief, thus strongly suggesting that belief is the mechanism behind the symptoms. *Distraction:* Distraction, or varying participants' attention to other stimuli, is an important principle of the NH-CBT movement retraining and is almost always the way to create this momentarily improved functioning. A distractor is previously selected collaboratively by therapist and participant (e.g., their favorite music) and is presented whilst participants engage in relevant physical movement. *Video recording/feedback*: Another important element of the NH-CBT movement retraining is video recording and immediate video feedback if the participant shows improved movement when distracted. Improved functioning seen on video feedback is framed as evidence that the “nocebo hypothesis” is valid: symptoms are not caused by structural neurological damage and their bodies are more capable than participants previously believed. More physical activity is then attempted, with further video feedback, and this treatment cycle is repeated, attempting more, and more complex activity as the participant improves. *Purposeful optimism:* Throughout treatment, therapists will be specifically instructed to portray as much optimism as possible to the participant about potential recovery, e.g., talking about how many people have got better using this treatment.	*Control movement retraining (2–23 sessions)*: The movement retraining part of the control intervention is based on a sequential motor learning approach proposed by a consensus recommendation for physiotherapy for functional motor disorders (16). The main rationale for movement retraining is that FNSD/PMD can be conceived of as unwanted patterns of movement that have been subconsciously programmed and learned, and are outside the person's control. It is assumed that these patterns cause symptoms and that symptoms are perpetuated by secondary changes. Movement can be retrained/“reprogrammed” by utilizing a sequential approach to re-establishing more normal movement patterns. *Assessment:* The assessment includes determining levels of falls risk, methods of keeping the person safe, observation of the impact of symptoms on posture and movement, exploring the nature of symptoms, evaluation of any potential compensatory strategies, and exploring possible secondary physical changes. *Sequential approach to restore normal movement patterns*: Elementary symptom free components of movement are established, and built on in successive stages to gradually reshape normal movement patterns. Another target is to change maladaptive compensatory habitual postures, movement patterns, and behaviors. Finally, secondary changes (e.g., physical changes such as deconditioning or changes of social/occupational roles) are addressed. Different movement retraining strategies are provided for different forms of functional motor symptoms in the treatment manual. Therapists are asked to avoid mentioning concepts such as nocebo/placebo effects, beliefs, or expectations. No visual feedback (neither video nor mirror feedback) is used. External distractors (e.g., music, irrelevant conversation) to vary participants' attention are not allowed. Purposeful optimism should not be part of therapists' approach.
	*NH-CBT relapse prevention (1 session)*: The treatment cycle is taken as far as possible in the final session. Therapist and participant collaboratively set a final movement goal that is as high as the participant wishes. The participant then tries to achieve it. The second part of the relapse prevention stage gives clear advice to people about what to do if symptoms re-emerge in future. For each individual person, a personalized behavioral experiment is devised that will prove to participants that their most feared neurological event is not happening. This experiment involves them reminding themselves that minor transient symptom, often referred to as “glitches”, are harmless and are just the subconscious expressing a momentary doubt via a nocebo effect.	*Control relapse prevention (1 session)*A self-management plan is introduced to the participant, including a summary of useful strategies that help to normalize movement, activity plans to address boom and bust patterns and how to progress activity, future goals, and what to do on difficult days and during periods of symptom exacerbation. Therapists are instructed to avoid making any suggestions that state or imply the role of illness beliefs.
5. Who provided	*Neurological assessment:* All neurologists involved in this trial will be employed on consultant level. They will receive training from the PI (MR) in person or by telephone. They will have been given suggestions by Stone (29) of how to explain FNSD/PMD to participants.	
	*NH-CBT psychoeducation*: clinical psychologist (PI: MR)	*Psychological input and supportive counseling*: clinical psychologist (other than PI)
	*NH-CBT movement retraining*: licensed rehabilitation professional (physiotherapist, occupational therapist, rehabilitation assistant), with a physiotherapist always having a role in developing the treatment plan (including safety management plan) and having oversight of the physical elements of the treatment	*Control movement retraining*: licensed rehabilitation professional (physiotherapist, occupational therapist, rehabilitation assistant), with a physiotherapist always having a role in developing the treatment plan (including safety management plan) and having oversight of the physical elements of the treatment
	Multi-disciplinary staff members delivering the initial educational or movement retraining intervention will have received training from the principal investigator (MR) which can take between 0.5 and 1 day (depending on the professional's level of experience). Each staff member will be trained either in NH-CBT or in the control intervention. We will use a brief checklist to ensure that the rehabilitation professional has demonstrated an understanding or proficiency in delivering the key ingredients of the intervention. All staff members involved in this trial will receive a comprehensive treatment manual. All professionals who deliver treatment within this trial are required to attend supervision frequently with a licensed supervisor experienced in CBT for individuals with functional neurological symptoms. The initial therapy session will be recorded. The supervisor will give immediate feedback to the therapists (of the control group in particular) in case they would expose participants to distraction techniques or interventions that address illness beliefs. At least one supervision session will be planned for every intervention participant treated.	
6. How	All sessions will be delivered as individual sessions in a face-to-face format as part of either an inpatient or outpatient treatment.	
7. Where	The majority of sessions will occur at the ISIS (Rehabilitation) Centre, Wakari Hospital, Dunedin, for both inpatient and outpatient treatment, although some sessions will occur in community settings for some participants, predominantly when they are in the latter stages of treatment.	
8. When and how much	1 neurology session (30–50 min) 1 initial session of education (60–90 min) Minimum 2 up to maximum 23 NH-CBT or control movement retraining sessions (30–120 min) over 8 weeks 1 session of relapse prevention (30 min) Participants can stop therapy before the 25 h limit if they (a) subjectively consider themselves to be fully recovered, (b) can mobilize at 5.0 km/h on a treadmill for 15 s, and (c) had at least one initial psychoeducation session and 2 sessions of movement retraining. These criteria are consistent with the suggestion by an international Functional Neurological Disorders Core Outcome Measure Group (35).	
9. Tailoring	The intervention is standardized by a treatment manual. The manual describes opportunities of how the education at the beginning can be tailored according to participants' individual functional motor symptoms and problems. The movement retraining session tasks and training will be adapted according to participants' individual symptoms. However the rehabilitation professionals are encouraged to focus on the key principles of the treatment.	
11. How well (planned)	All therapists in this trial must adhere to a therapy manual and receive training in the treatment content and procedure. Therapists are required to attend supervision frequently with a licensed supervisor experienced in CBT for individuals with functional neurological symptoms. Treatment fidelity will be assessed as follows: All initial therapy sessions will be audio-recorded. A checklist of key treatment objectives and principles will be used to assess treatment fidelity and its two components, therapist adherence and treatment specificity, for both the NH-CBT and the active control intervention. We will assess therapist competence with a validated rating scale [e.g., Cognitive Therapy Scale (36)]. Session records of a randomly selected sample (30% of the intention-to-treat sample) will be rated by two independent, blinded raters.	

*Item 10 (Modifications: If the intervention was modified during the course of the study, describe the changes) and 12 (How well [actual]: If intervention adherence or fidelity was assessed, describe the extent to which the intervention was delivered as planned) of the TIDieR Checklist do not apply to our trial and are omitted from this table*.

#### Key Strategies of and Differences Between NH-CBT and the Active Control Treatment

##### NH-CBT

The NH-CBT program builds on the predictive coding theory of FNSD/PMD (20). It is assumed that participants' motor symptoms can be explained by a strong mental representation of their body or parts of their body as neurologically damaged or diseased. The key component of this treatment is to retrain participants' symptom processing and to reduce the strength of this mental representation. The following four key strategies of NH-CBT are essential to address the four basic treatment principles mentioned earlier:

- To help participants to understand the role of their “priors” or beliefs as mechanisms causing their symptoms and to remind them about the neurological evidence for a lack of neurological damage or disease (i.e., what is not causing their symptoms), the concept of the “nocebo effect” is explained and used to inform a cognitive behavioral formulation which explains their symptoms. The psychoeducation component of NH-CBT shall provide the individual with a new, alternative belief (i.e., what is causing their symptoms).
- The movement retraining strategies are used in the sense of a behavioral experiment. They are essential to provide the participants with new and precise “motor input” to lower the precision and strength of their abnormal prior beliefs. The therapist and participant will continue reflecting on the change of “priors” and beliefs during the movement retraining as being the only possible mechanism behind any improvement.
- This process is video-recorded, and feedback is promptly given to the participant that any normalized movement is evidence confirming the nocebo explanation for their symptoms and disconfirming the presence of neurological damage.
- To divert participants' attention and to lower the precision-increasing effect on abnormal priors, they have to be distracted while they practice movements.

##### Supportive Counseling and Movement Retraining (Active Control Intervention)

The control intervention was designed to control for the non-specific effects of (a) education about symptoms, (b) emotional support from a therapist, and (c) movement retraining. For (a), the psychologist will reassure the participant that symptoms are not caused by structural damage or a degenerative process, however there will be no conversation about beliefs or expectations being an important factor in the development and maintenance of functional motor symptoms. The concept of a nocebo effect will not be mentioned. The psychologist will mainly repeat the explanation of FNSD/PMD by Stone (29) that will have already been provided by the neurologist during the initial neurological assessment. For (b) the psychologist will provide supportive counseling about the participants' symptoms and associated distress and everyday impact, and strategies to manage distress such as relaxation exercises. For (c), motor symptoms will be conceived of as learnt patterns of movement that are outside participants' control. The movement retraining will attempt to re-establish normal movement patterns, but without video feedback and distraction techniques.

The active control intervention will not address any of the key treatment strategies of NH-CBT. The active control intervention will differ from the NH-CBT regarding the following aspects:

- It will not include any discussion of the concept of the “nocebo effect” or the role of beliefs as mechanisms of functional motor symptoms.
- The movement retraining will not be used in the sense of a behavioral experiment, but as a systematic attempt to re-establish normal movement.
- It will not involve any means of visual feedback.
- It will not use any engrossing, external distractors, such as music, video, video games, irrelevant conversation, or counting. The only redirecting of attention will involve a suggestion that the participant focus on a point in space, instead of on their bodies.

### Outcomes and Assessments

The choice of primary and secondary outcome measures was based on the recommendations of the Functional Neurological Disorder-Core Outcome Measures Group (35) and the European Network of Somatic Symptom Disorders (38). The *primary outcome* and *endpoint* will be the 36-item Short Form Health Survey (SF-36) Physical Functioning subscale at 8-week follow-up (39, 40). DSM-5 has shifted the focus of classification from the assessment of somatic symptoms to the consideration of concurrent psychosocial impacts (1). This 10-item Physical Functioning subscale assesses a range of severe and minor physical limitations during a typical day. Health-related quality of life is classified as one of the most relevant outcome domains in clinical trials with individuals with persistent somatic symptoms and FNSD/PMD in particular (38). The internal consistency is high (Cronbach's alpha = 0.90) (40). All *secondary outcome measures* are summarized in Table 2. The use of the EuroQol-5 Dimension Questionnaire (EQ-5D-5L) will allow us to compare our intervention and control treatment regarding different health economic indices (e.g., Disability-Adjusted Life Year [DALYs] or Quality-Adjusted Life Years [QALYs]). Different dimensions of healthcare utilization, assessed with the Client Service Receipt Inventory (CSRI) and from participants' electronic health records, will allow us to measure further cost signals.

**Table 2:** Assessments, outcomes, and measures.

**Outcome domain**	**Measure**	**B**	8-w FU^*^	**16-w FU**	**24-w FU**
**Primary outcome measure**					
Physical functioning	Short Form-36 Health Questionnaire (SF-36), Physical Functioning subscale (39)	x	x	x	x
**Secondary outcome measures**					
**Somatic symptoms**					
Functional motor symptoms	Simplified Functional Movement Disorder Rating Scale (S-FMDRS)^*^ (30)	x	x	x	x
Fatigue	Short Form-36 Health Questionnaire (SF-36), Vitality subscale (39)	x	x	x	x
Somatic symptom severity	Patient Health Questionnaire-15 (PHQ-15)	x	x	x	x
**Illness consequences**					
Mobility	10 m-Walk Test^*^ (32)	x	x	x	x
	Functional Mobility Scale (FMS)^*^ (31)	x	x	x	x
Symptom disability	Modified Pain Disability Index (PDI) (41, 42)	x	x	x	x
Health-related quality of life	Short Form-36 Health Questionnaire (SF-36) (39)	x	x	x	x
Quality of Life	EuroQol-5D (EQ-5D-5L) (43)	x	x	x	x
Health care utilization (past 3 months)	Item 7 and 8 of the modified Client Service Receipt Inventory (CSRI) for chronic pain patients (44, 45)	x		x	
Health care utilization	Participants' electronic health records	x		x	
**Psychological features**					
Anxiety	Generalized Anxiety Scale-7 (GAD-7) (46)	x	x	x	x
Depression	Patient Health Questionnaire-9 (PHQ-9) (47)	x	x	x	x
Catastrophic thinking about symptoms	Symptom Catastrophizing Scale (SCS) (48)	x	x	x	x
Illness perception	Brief Illness Perception Questionnaire (BIPQ) (49)	x	x	x	x
Dissociation	Brief Dissociative Experiences Scale (B-DES) (50, 51)	x	x	x	x
**Participant satisfaction**					
Therapeutic alliance	Work Alliance Inventory—Short Form (WAI-SR) (52)		x		x
Participant's perception of change	Clinical Global Impression Scale of Improvement (CGI-I) (53)		x	x	x
**Adverse events**					
Adverse events in therapy	Inventory for the Assessment of Negative Effects of Psychotherapy (INEP) (54)		x		x
Adverse events in therapy	Severity Assessment Code rating for adverse event reporting (SAC)^†^(33)				

* *Blinded assessor rating*.

† *The SAC will be applied during the 8 weeks of NH-CBT in case of serious adverse events*.

### Data Management and Statistical Methods

#### Data Management and Dropout From Study

In order to enter all variables defined in the study protocol, standardized electronic case report forms will be prepared and administered. All questionnaires will be set up electronically and administered on a laptop. Data entry will only allow values in a valid range. Prior to data analyses, data will be checked for plausibility.

In this trial two different kinds of dropout will be defined: a study dropout and therapy dropout. A study dropout is defined as a participant who misses at least one of the main assessments (baseline, 8- or 16-week follow-up, see Figure 1). A therapy dropout is defined as a participant

- who stops therapy before the initial session and a minimum of two movement retraining sessions,
- who stops therapy after the minimum number of sessions but before the therapy goal has been reached (typical reasons: low therapy adherence, external factors such as moving away, etc.), or
- who no longer meets eligibility criteria (e.g., due to change in regimen of psychopharmacotherapy).

The reason for dropout must be documented by the therapist. Even if participants drop out of the treatment or become non-eligible during the course of the therapy, they will be encouraged to complete further follow-up assessments.

#### Sample Size, Power Calculations, and Interim Analyses

The power analysis for our trial was run with G^*^Power 3.1.9.4. In a previous trial, a physiotherapy for functional motor symptoms reached a moderate effect (*f* = 0.33) at 6-month follow-up on the SF-36 Physical Functioning subscale, our primary outcome (23). For this trial we conservatively assume a small group^*^time interaction effect (*f* = 0.10) under consideration of our three measurements and a test-retest reliability of *r* = 0.5. A power analysis for a mixed-effect ANOVA with an alpha error probability of 0.05 and a power of 0.80 results in an optimal total sample size of 164. A dropout rate of 5% is estimated based on our clinical experience and a recently completed trial that evaluated physiotherapy for individuals with FNSD (23). Accordingly a sample size of 172 participants is planned to be recruited.

We will, however, allow eight interim analyses every 22nd patient, conducted by a person who is independent of the day-to-day activities of the trial. Using the Lan-DeMets alpha spending function (55) we have gained appropriate boundaries or *p*-values for each interim analysis that would indicate a necessary stop and would allow an early conclusion of the trial (see Table 3). We used R 4.0.2 (56) and the ldbounds package (57) to gain Lan-DeMets boundaries. This approach helps to control for alpha-error accumulation with repeated interim analyses.

**Table 3:** *P*-values (based on Lan-DeMets alpha spending function) indicating a necessary stop of the trial and needed sample size at each interim analysis.

**Interim analysis**	**Sample size**	***P*-value**
1	22	0.00000003
2	44	0.00009
3	66	0.001
4	88	0.005
5	110	0.013
6	133	0.024
7	155	0.036
8	172	0.050

#### Statistical Analysis

Analyses will be based on intention-to-treat principles and will be carried out at the end of the last follow-up assessment. We will apply a linear mixed-effect model with baseline adjustment with main effects (fixed effect) for time (level 1, contrasts to baseline at 8- and 16-week follow-up) and group (level 2), group-by-time cross-level interaction term (8- and 16-week follow-up), and a generalized covariance matrix to account for dependency among observations. In order to deal with missing values, maximum likelihood estimation will be applied. Patterns of missing values will be assumed to be random. The outcomes of interest will be the group-by-time interaction at week 8 (hypothesis 1 and 2), and at week 16 (hypothesis 3). Effect sizes (Cohen's d) with 95% CI of changes between baseline and 8- and 16-week follow-up, as well as of between-group differences at 8- and 16-week follow-up will be investigated. Further sensitivity analyses will be conducted depending on the dropout rates and pattern of dropout reasons. For this purpose the linear mixed-effect models described above will be rerun for defined subgroups of the total sample (e.g., for participants with complete datasets only). To test for moderation (exploratory research question) we will add the moderator variable to the linear mixed-effect model described above (level 1: time; level 2: group) and will analyze a 3-level interaction. A significance level of 5% will apply to all analyses. Statistical analyses will be conducted with SPSS.

A limitation of group analyses is that it provides no information about the relative proportions of participants who did or did not respond to a treatment, which is based on a set standard. In accordance with recommendations by the Initiative on Methods, Measurement, and Pain Assessment in Clinical Trials (58), we will compare the distribution of participants with an improvement of at least 30 or 50%, no change, or deterioration on the primary outcome between the study groups with χ^2^-tests. Groups will be compared regarding adverse therapy effects, measured with the Inventory for the Assessment of Negative Effects of Psychotherapy, with independent sample *t*-tests and χ^2^-tests. To assess interrater reliability of the treatment fidelity ratings, intraclass coefficients (two-way random model with measure of agreement) for each item will be computed.

## Discussion

The central aim of this RCT is to examine the efficacy of an innovative interdisciplinary treatment for individuals with functional motor symptoms. We aim to combine CBT- and physiotherapy-based strategies not just in a cumulative sense but by intertwining their mechanisms and by embedding them within a singular coherent theoretical framework, the predictive coding model of FNSD (20). For example, we will use a movement retraining to boost the effect of psychoeducation and cognitive restructuring to establish new functional symptom beliefs by regularly reflecting with the participants on the role of beliefs during the movement retraining and providing the participant with the behavioral evidence of normal and healthy movement.

We plan a randomized trial implementing an active control intervention that is designed to control for non-specific effects of education, emotional support, and movement retraining. A strength of this trial is that we will be able to gain a controlled assessment at the end of treatment and 8 weeks later. Another strength of this design is the mixed method approach, including clinician-rated, observer-based, and patient-reported outcomes. Blinded assessors will help to reduce the risk of detection bias.

Besides the strengths, we anticipate several limitations. We have already discussed risks of bias and have implemented tools to reduce these risks. There are additional challenges that we anticipate with implementing the study procedure. First, recruiting from one hospital can limit the representativeness of our sample and can hamper the potential to gain the optimal sample size. The institution where we will conduct our trial covers the neurological rehabilitation of a substantial part of the NZ population, which will ensure our sample is largely representative for the region. We first want to ensure the feasibility of our trial at this site before considering the potential to extend the trial to at least one other site. With a priori planned interim analyses, we will regularly check if the conservative estimation of our optimal sample size will need correction. Second, the design of our active control intervention has several advantages mentioned above. Nonetheless, we have to expect indirect therapeutic effects of our active control treatment (e.g., incidental change in beliefs after re-establishing a normal movement). Even accounting for the active control treatment being potentially less effective than NH-CBT, it seems likely that the mean difference between groups will be small. Our power analysis therefore builds on a conservatively estimated small between-group effect. When designing the control intervention, we also had to consider the pragmatic fact that a treatment-as-usual or a waitlist control group would not have been feasible, because our institution is the only one covering neurorehabilitation for patients from a bigger district in New Zealand, implying limited resources and long waitlists for receiving treatment. Third, another pragmatic limitation is that we have to accept participants in the inpatient and outpatient settings as well as participants of older ages. We will apply statistical strategies to control for the type of treatment setting in our data analysis. We will include older participants only if motor symptoms are considered highly likely to be functional and not related to age-related degenerative processes. Fourth, our 8-week follow-up window after the end of treatment is very short. Again, due to pragmatic reasons associated with delivering both treatments in the context of a clinical service, we would not be able to let our control participants wait for a longer period than this before they receive NH-CBT. At the same time, we consider the *controlled* follow-up assessment as an important strength of our study design. Finally, we will implement several methods to ensure treatment fidelity (e.g., frequent supervision, audio-recording the initial therapy session, and feedback to therapists). However, we will not be able to completely exclude the risk of therapists deviating from our rules of treatment fidelity and exposing participants to distraction techniques or interventions that address illness beliefs.

NH-CBT is a strongly theory-driven treatment concept, combining the two first-line interventional approaches for FNSD/PMD in a unique way. Its interdisciplinary character, involving neurologists, clinical psychologists, physiotherapists, occupational therapists, and other rehabilitation professionals is in accordance with a biopsychosocial understanding of persistent physical symptoms. This stands in contrast to a more reductionistic biomedical model and arguably better addresses the multidimensional etiopathogenesis of a complex phenomenon such as functional motor symptoms. We see much potential to improve the treatment outcome for patients with complex conditions such as FNSD in formulating links between different disciplines into a coherent, theory-driven, truly interdisciplinary approach. Results from previous research focusing on monodisciplinary approaches have not been convincing. For example, a recently published RCT showed no effect of a sophisticated CBT treatment for patients with functional epileptic seizures on the frequency and severity of seizures and mostly small effects on secondary outcomes compared with a treatment as usual (59).

First results from a case series examining the effects of NH-CBT show promising effects (27). The sensible next step is to investigate the efficacy of NH-CBT in a randomized trial and to gain knowledge about how well the intervention's effect can be sustained and by which variables it is moderated. If demonstrated to have efficacy, we would then prioritize an evaluation of its effectiveness and feasibility nationally and internationally, with the aim of implementing NH-CBT into routine practice worldwide. We anticipate challenges associated with implementing an interdisciplinary treatment that requires a whole team to be committed to a mutual therapeutic understanding. However, the experience at the rehabilitation center where NH-CBT originated has been that this was not a considerable impediment in the context of an existing interdisciplinary approach to neurorehabilitation. Countries such as New Zealand and Australia are often progressive in implementing treatment for chronic conditions in interdisciplinary treatment settings, e.g., the National Pain Strategy in Australia (60). A successful trial has the capacity to transform the lives of individuals living with functional neurological symptoms and substantially reduce financial burdens on healthcare systems around the world.

## Ethics Statement

The studies involving human participants were reviewed and approved by the Southern Health and Disability Ethics Committee of New Zealand. The patients/participants will provide their written informed consent to participate in this study.

## Author Contributions

MR: conceptualization, data curation, formal analysis, funding acquisition, investigation, methodology, project administration, resources, validation, visualization, roles/writing—original draft, and writing—review and editing. DW: conceptualization, formal analysis, methodology, supervision, validation, visualization, and writing—review and editing. MK: conceptualization, data curation, formal analysis, funding acquisition, methodology, project administration, resources, supervision, validation, visualization, roles/writing—original draft, and writing—review and editing. All authors contributed to the article and approved the submitted version.

## Conflict of Interest

The authors declare that the research was conducted in the absence of any commercial or financial relationships that could be construed as a potential conflict of interest.

## Abbreviations

CBT: cognitive behavioral therapy
DSM-5: 5th revision of the Diagnostic and Statistical Manual of Mental Disorders
FNSD: Functional Neurological Symptom Disorder
NH-CBT: Nocebo-Hypothesis Cognitive Behavioral Therapy
PMD: psychogenic movement disorder
RCT: randomized controlled trial
SAE: serious adverse events.

## References

[1] American Psychiatric Association Diagnostic and Statistical Manual of Mental Disorders. Fifth edition. DSM-5. Washington, DC: APA (2013). 10.1176/appi.books.9780890425596

[2] Gupta A Lang AE. Psychogenic movement disorders. Curr Opin Neurol. (2009) 22:430–6. 10.1097/WCO.0b013e32832dc169 19542886

[3] Carson A Lehn A Epidemiology. In: Hallett M, Stone J, Carson A, editors. Functional Neurologic Disorders, Vol 139 of the Handbook of Clinical Neurology Series. Amsterdam: Elsevier (2016). p. 47–60. 10.1016/B978-0-12-801772-2.00005-9

[4] Carson A Stone J Hibberd C Murray G Duncan R Coleman R. Disability, distress and unemployment in neurology outpatients with symptoms 'unexplained by organic disease'. J Neurol Neurosurg Psychiatry. (2011) 82:810–3. 10.1136/jnnp.2010.220640 21257981

[5] Anderson KE Gruber-Baldini AL Vaughan CG Reich SG Fishman PS Weiner WJ. Impact of psychogenic movement disorders versus Parkinson's on disability, quality of life, and psychopathology. Movement Disord. (2007) 22:2204–9. 10.1002/mds.21687 17876850

[6] Feinstein A Stergiopoulos V Fine J Lang AE. Psychiatric outcome in patients with a psychogenic movement disorder. Neuropsychiatry Neuropsychol Behav Neurol. (2001) 14:169–76. 11513100

[7] Barsky AJ Orav EJ Bates DW. Somatization increases medical utilization and costs independent of psychiatric and medical comorbidity. Arch Gen Psychiatry. (2005) 62:903–10. 10.1001/archpsyc.62.8.903 16061768

[8] Stone J Carson A Duncan R Roberts R Warlow C Hibberd C. Who is referred to neurology clinics?-The diagnoses made in 3781 new patients. Clin Neurol Neurosurg. (2010) 112:747–51. 10.1016/j.clineuro.2010.05.011 20646830

[9] Carson AJ Best S Postma K Stone J Warlow C Sharpe M. The outcome of neurology outpatients with medically unexplained symptoms: a prospective cohort study. J Neurol Neurosurg Psychiatry. (2003) 74:897–900. 10.1136/jnnp.74.7.897 12810775 PMC1738573

[10] Rosebush PI Mazurek MF. Treatment of conversion disorder in the 21st century: have we moved beyond the couch? Curr Treat Options Neurol. (2011) 13:255–66. 10.1007/s11940-011-0124-y 21468672

[11] Espay AJ Aybek S Carson A Edwards MJ Goldstein LH Hallett M. Current concepts in diagnosis and treatment of functional neurological disorders. JAMA Neurol. (2018) 75:1132–41. 10.1001/jamaneurol.2018.1264 29868890 PMC7293766

[12] Feinstein A. Conversion disorder. Continuum. (2018) 24:861–72. 10.1212/CON.0000000000000601 29851882

[13] Lafrance WCJr Friedman JH. Cognitive behavioral therapy for psychogenic movement disorder. Movement Disord. (2009) 24:1856–7. 10.1002/mds.22683 19562779

[14] Dallocchio C Tinazzi M Bombieri F Arno N Erro R. Cognitive behavioural therapy and adjunctive physical activity for functional movement disorders (conversion disorder): a pilot, single-blinded, randomized study. Psychother Psychosom. (2016) 85:381–3. 10.1159/000446660 27744440

[15] Czarnecki K Thompson JM Seime R Geda YE Duffy JR Ahlskog JE. Functional movement disorders: successful treatment with a physical therapy rehabilitation protocol. Parkinsonism Relat Disord. (2012) 18:247–51. 10.1016/j.parkreldis.2011.10.011 22113131

[16] Nielsen G Stone J Matthews A Brown M Sparkes C Farmer R. Physiotherapy for functional motor disorders: a consensus recommendation. J Neurol Neurosurg Psychiatry. (2015) 86:1113–9. 10.1136/jnnp-2014-309255 25433033 PMC4602268

[17] Mccormack R Moriarty J Mellers JD Shotbolt P Pastena R Landes N. Specialist inpatient treatment for severe motor conversion disorder: a retrospective comparative study. J Neurol Neurosurg Psychiatry. (2014) 85:893–8. 10.1136/jnnp-2013-305716 24124043

[18] Jacob AE Kaelin DL Roach AR Ziegler CH Lafaver K. Motor Retraining (MoRe) for functional movement disorders: outcomes from a 1-week multidisciplinary rehabilitation program. PM&R. (2018) 10:1164–72. 10.1016/j.pmrj.2018.05.011 29783067

[19] Jordbru AA Smedstad LM Klungsoyr O Martinsen EW. Psychogenic gait disorder: a randomized controlled trial of physical rehabilitation with one-year follow-up. J Rehabil Med. (2014) 46:181–7. 10.2340/16501977-1246 24248149

[20] Edwards MJ Adams RA Brown H Parees I Friston KJ. A Bayesian account of 'hysteria'. Brain. (2012) 135:3495–512. 10.1093/brain/aws129 22641838 PMC3501967

[21] Fobian AD Elliott L. A review of functional neurological symptom disorder etiology and the integrated etiological summary model. J Psychiatry Neurosci. (2019) 44:8–18. 10.1503/jpn.170190 30565902 PMC6306282

[22] Friston KJ. A theory of cortical responses. Philoso Transact R Soc B-Biol Sci. (2005) 360:815–36. 10.1098/rstb.2005.1622 15937014 PMC1569488

[23] Nielsen G Buszewicz M Stevenson F Hunter R Holt K Dudziec M. Randomised feasibility study of physiotherapy for patients with functional motor symptoms. J Neurol Neurosurg Psychiatry. (2017) 88:484–90. 10.1136/jnnp-2016-314408 27694498

[24] Colloca L Miller FG. Role of expectations in health. Curr Opin Psychiatry. (2011) 24:149–55. 10.1097/YCO.0b013e328343803b 21248640

[25] Kennedy WP. The nocebo reaction. Med World. (1961) 95:203–5. 13752532

[26] Petrie KJ Rief W. Psychobiological mechanisms of placebo and nocebo effects: pathways to improve treatments and reduce side effects. Ann Rev Clin Psychol. (2018) 70:12.11-12.27. 10.1146/annurev-psych-010418-102907 30110575

[27] Richardson M Isbister G Nicholson B. A novel treatment protocol (Nocebo Hypothesis Cognitive Behavioural Therapy; NH-CBT) for functional neurological symptom disorder/conversion disorder: a retrospective consecutive case series. Behav Cogn Psychother. (2018) 46:497–503. 10.1017/S1352465817000832 29463338

[28] Chan AW Tetzlaff JM Altman DG Laupacis A Gotzsche PC Krleza-Jeric K. SPIRIT 2013 statement: defining standard protocol items for clinical trials. Ann Intern Med. (2013) 158:200–7. 10.7326/0003-4819-158-3-201302050-00583 23295957 PMC5114123

[29] Stone J. Functional neurological disorders: the neurological assessment as treatment. Neurophysiol Clinique-Clin Neurophysiol. (2014) 44:363–73. 10.1016/j.neucli.2014.01.002 25306077

[30] Nielsen G Ricciardi L Meppelink AM Holt K Teodoro T Edwards M. A simplified version of the psychogenic movement disorders rating scale: the Simplified Functional Movement Disorders Rating Scale (S-FMDRS). Movement Disord Clin Pract. (2017) 4:710–6. 10.1002/mdc3.12475 30363505 PMC6174502

[31] Graham HK Harvey A Rodda J Nattrass GR Pirpiris M The Functional Mobility Scale (FMS). J Pediatric Orthopaed. (2004) 24:514–20. 10.1097/01241398-200409000-00011 15308901

[32] Rossier P Wade DT. Validity and reliability comparison of 4 mobility measures in patients presenting with neurologic impairment. Arch Phys Med Rehabil. (2001) 82:9–13. 10.1053/apmr.2001.9396 11239279

[33] New South Wales Health Department Severity Assessment Code (SAC) Matrix. Sydney, NSW: NSW Health (2005).

[34] Health Quality and Safety Commission New Zealand Severity Assessment Code (SAC) Rating and Triage Tool for Adverse Event Reporting. Wellington, NZ (2017).

[35] Pick S Anderson DG Asadi-Pooya AA Aybek S Baslet G Bloem BR. Outcome measurement in functional neurological disorder: a systematic review and recommendations. J Neurol Neurosurg Psychiatr. (2020) 91:638–49. 10.1136/jnnp-2019-322180 32111637 PMC7279198

[36] Young J Beck A Cognitive Therapy Scale: Rating Manual. Philadelphia, PA: Center for Cognitive Therapy (1980). 10.1037/t00834-000

[37] Hoffmann TC Glasziou PP Boutron I Milne R Perera R Moher D. Better reporting of interventions: template for intervention description and replication (TIDieR) checklist and guide. BMJ. (2014) 348:g1687. 10.1136/bmj.g1687 24609605

[38] Rief W Burton C Frostholm L Henningsen P Kleinstäuber M Kop WJ. Core outcome domains for clinical trials on somatic symptom disorder, bodily distress disorder and functional somatic syndromes: EURONET-SOMA recommendations. Psychosom Med. (2017) 79:1008–15. 10.1097/PSY.0000000000000502 28691994

[39] Ware JE Snoww KK Kosinski MA Gandek BG SF36 Health Survey: Manual and Interpretation Guide. Boston, MA: The Health Institute, New England Medical Centre (1993).

[40] Jenkinson C Wright L Coulter A. Criterion validity and reliability of the SF-36 in a population-sample Qual Life Res. (1994) 3:7–12. 10.1007/BF00647843 8142947

[41] Tait RC Chibnall JT Krause S. The pain disability index - psychometric properties. Pain. (1990) 40:171–82. 10.1016/0304-3959(90)90068-O 2308763

[42] Mewes R Rief W Stenzel N Glaesmer H Martin A Brähler E What is “normal” disability? An investigation of disability in the general population. Pain. (2009) 142:36–41. 10.1016/j.pain.2008.11.007 19147292

[43] Williams A Euroqol - a new facility for the measurement of health-related quality of life. Health Policy. (1990) 16:199–208. 10.1016/0168-8510(90)90421-9 10109801

[44] Beecham J Knapp M Mcgilloway S Donnelly M Kavanagh S Fenyo A. The cost-effectiveness of community care for adults with learning disabilities leaving long-stay hospital in Northern Ireland. J Intell Disabil Res. (1997) 41:30–41. 10.1111/j.1365-2788.1997.tb00674.x 9089457

[45] Sleed M Eccleston C Beecham J Knapp M Jordan A. The economic impact of chronic pain in adolescence: methodological considerations and a preliminary costs-of-illness study. Pain. (2005) 119:183–90. 10.1016/j.pain.2005.09.028 16297552

[46] Spitzer RL Kroenke K Williams JBW Loewe B A brief measure for assessing generalized anxiety disorder - the GAD-7. Arch Intern Med. (2006) 166:1092–7. 10.1001/archinte.166.10.1092 16717171

[47] Kroenke K Spitzer RL Williams JBW. The PHQ-9 - Validity of a brief depression severity measure. J Gen Intern Med. (2001) 16:606–13. 10.1046/j.1525-1497.2001.016009606.x 11556941 PMC1495268

[48] Moore E Adams H Ellis T Thibault P Sullivan MJL. Assessing catastrophic thinking associated with debilitating mental health conditions. Disabil Rehabil. (2018) 40:317–22. 10.1080/09638288.2016.1254283 27866430

[49] Broadbent E Petrie KJ Main J Weinman J. The brief illness perception questionnaire. J Psychosom Res. (2006) 60:631–7. 10.1016/j.jpsychores.2005.10.020 16731240

[50] Bernstein EM Putnam FW. Development, reliability, and validity of a dissociation Scale. J Nerv Mental Dis. (1986) 174:727–35. 10.1097/00005053-198612000-00004 3783140

[51] Dalenberg C Carlson E New versions of the Dissociative Experiences Scale: The DES-R (revised) and the DES-B (brief). In: Annual Meeting of the International Society for Traumatic Stress Studies. Montreal, QC (2010).

[52] Hatcher RL Gillaspy JA Development and validation of a revised short version of the Working Alliance Inventory. Psychother Res. (2006) 16:12–25. 10.1080/10503300500352500

[53] Sharpe M Walker J Williams C Stone J Cavanagh J Murray G. Guided self-help for functional (psychogenic) symptoms A randomized controlled efficacy trial. Neurology. (2011) 77:564–72. 10.1212/WNL.0b013e318228c0c7 21795652 PMC3149156

[54] Ladwig I Rief W Nestoriuc Y What are the risks and side effects to psychotherapy? -Development of an Inventory for the Assessment of Negative Effects of Psychotherapy (INEP) [Welche Risiken und Nebenwirkungen hat Psychotherapie? – Entwicklung des Inventars zur Erfassung Negativer Effekte von Psychotherapie (INEP)] Verhaltenstherapie. (2014) 24:252–63. 10.1159/000367928

[55] Lan KKG Demets DL Discrete sequential boundaries for clinical trials Biometrika. (1983) 70:659–63. 10.1093/biomet/70.3.659

[56] R. Core Team R: A Language and Environment for Statistical Computing. Vienna: R Foundation for Statistical Computing (2013).

[57] Casper C Perez OA ldbounds: Lan-DeMets Method for Group Sequential Boundaries. R package version *1.1-1.1* Available online at: https://cran.r-project.org/web/packages/ldbounds/ldbounds.pdf (accessed November 13, 2020).

[58] Dworkin RH Turk DC Farrar JT Haythornthwaite JA Jensen MP Katz NP. Core outcome measures for chronic pain clinical trials: IMMPACT recommendations. Pain. (2005) 113:9–19. 10.1016/j.pain.2004.09.012 15621359

[59] Goldstein LH Robinson EJ Mellers JDC Stone J Carson A Reuber M. Cognitive behavioural therapy for adults with dissociative seizures (CODES): a pragmatic, multicentre, randomised controlled trial. Lancet Psychiatry. (2020) 7:491–505. 10.1016/S2215-0366(20)30128-0 32445688 PMC7242906

[60] National Pain Summit National Pain Strategy. Pain Management for All Australians. (2010). Available online at: https://www.chronicpainaustralia.org.au/files/PainStrategy2010Final.pdf (accessed 22 March, 2019).

